# NMNAT Proteins that Limit Wallerian Degeneration Also Regulate Critical Period Plasticity in the Visual Cortex

**DOI:** 10.1523/ENEURO.0277-18.2018

**Published:** 2019-01-18

**Authors:** Mariska van Lier, Laura Smit-Rigter, Roos Krimpenfort, M. Hadi Saiepour, Emma Ruimschotel, Willem Kamphuis, J. Alexander Heimel, Christiaan N. Levelt

**Affiliations:** 1 Molecular Visual Plasticity Group, Netherlands Institute for Neuroscience, Meibergdreef 47, 1105 BA Amsterdam, The Netherlands; 2 Cortical Structure and Function Group, Netherlands Institute for Neuroscience, Meibergdreef 47, 1105 BA Amsterdam, The Netherlands; 3 Netherlands Institute for Neuroscience, Meibergdreef 47, 1105 BA Amsterdam, The Netherlands; 4 Department of Molecular and Cellular Neurobiology, Center for Neurogenomics and Cognitive Research, Vrije Universiteit Amsterdam, De Boelelaan 1105, 1081 HV Amsterdam, The Netherlands

**Keywords:** axon, cortex, critical period, plasticity, Wallerian degeneration

## Abstract

Many brain regions go through critical periods of development during which plasticity is enhanced. These critical periods are associated with extensive growth and retraction of thalamocortical and intracortical axons. Here, we investigated whether a signaling pathway that is central in Wallerian axon degeneration also regulates critical period plasticity in the primary visual cortex (V1). Wallerian degeneration is characterized by rapid disintegration of axons once they are separated from the cell body. This degenerative process is initiated by reduced presence of cytoplasmic nicotinamide mononucleotide adenylyltransferases (NMNATs) and is strongly delayed in mice overexpressing cytoplasmic NMNAT proteins, such as Wld^S^ mutant mice producing a UBE4b-NMNAT1 fusion protein or NMNAT3 transgenic mice. Here, we provide evidence that in Wld^S^ mice and NMNAT3 transgenic mice, ocular dominance (OD) plasticity in the developing visual cortex is reduced. This deficit is only observed during the second half of the critical period. Additionally, we detect an early increase of visual acuity in the V1 of Wld^S^ mice. We do not find evidence for Wallerian degeneration occurring during OD plasticity. Our findings suggest that NMNATs do not only regulate Wallerian degeneration during pathological conditions but also control cellular events that mediate critical period plasticity during the physiological development of the cortex.

## Significance Statement

Different forms of axon degeneration occur during development and neurodegenerative processes. A good understanding of the molecular and cellular events that regulate these forms of axon degeneration is essential to selectively modulate them for therapeutic purposes. This study shows that genes thought to be selectively involved in pathologic axon degeneration are also involved in developmental plasticity, implying that these events are molecularly less separable than previously assumed.

## Introduction

Many regions of the brain go through defined phases of development, known as critical periods, during which experience-dependent plasticity is enhanced. Plasticity deficits during these critical periods have lifelong consequences. Understanding the molecular and cellular mechanisms that control critical period closure may help the development of approaches to reopen critical periods at a later age for therapeutic purposes. The most informative model to study critical period regulation has been ocular dominance (OD) plasticity in mouse primary visual cortex (V1). OD plasticity occurs during development to optimize binocular vision, matching the inputs of both eyes and, if this is not possible, biasing V1 toward inputs from the eye providing the most reliable inputs. When one eye is monocularly deprived (MD) for several days, developing V1 adjusts to this situation by rearranging thalamocortical and corticocortical connectivity. This results in a shift of responsiveness of V1 neurons toward the open eye (Gordon and Stryker, 1996; Antonini et al., 1999; Haruta and Hata, 2007). In adult mice in which the critical period in V1 is closed, prolonged MD causes a weaker and less permanent shift (Sawtell et al., 2003; Hofer et al., 2006; Heimel et al., 2007; Lehmann and Löwel, 2008; Sato and Stryker, 2008). Over the last two decades, this model revealed that maturation of inhibitory innervation is the dominant mechanism regulating critical period closure. However, various other cellular events have been implicated in critical period closure, including excitatory synapse maturation (Tropea et al., 2010; Bochner et al., 2014; Stephany et al., 2014; Huang et al., 2015; Jenks et al., 2017), extracellular matrix development (Pizzorusso et al., 2002), CREB transcription (Pham et al., 2004; Tognini et al., 2011), neuromodulatory inputs (Bear and Singer, 1986; Morishita and Hensch, 2008; Vetencourt et al., 2008), and declining neurite growth and retraction (McGee et al., 2005; Syken et al., 2006).

In a study analyzing changes in the synaptic proteome during development and OD plasticity of V1, several proteins involved in Wallerian axon degeneration were identified, whose expression levels changed around the time of critical period closure (Dahlhaus et al., 2011). Wallerian degeneration typically occurs after the nerve is separated from the cell body after injury (Kerschensteiner et al., 2005; Coleman and Freeman, 2010; Pease and Segal, 2014; Geden and Deshmukh, 2016; Gerdts et al., 2016). Because retraction of thalamocortical and intracortical axons also occurs in V1 during OD plasticity, we were intrigued by the possibility that the signaling pathway that mediates Wallerian degeneration also regulates axonal reorganization during OD plasticity.

During Wallerian degeneration, disintegration of the axon starts by breakdown of the cytoskeleton, followed by degradation of the myelin sheath (Vargas and Barres, 2007; Gilley and Coleman, 2010). Nicotinamide mononucleotide adenylyltransferases (NMNATs), key proteins in the nicotinamide adenine dinucleotide (NAD) biosynthetic pathway, are implicated in regulating Wallerian degeneration. Wallerian degeneration is slowed down considerably in Wld^S^ mice, which carry a spontaneous mutation causing overexpression of an axonally targeted UBE4b-NMNAT1 fusion protein (Coleman et al., 1998; Lunn et al., 1989; Lyon et al., 1993; Conforti et al., 2000, 2007, 2009). NMNAT3, which is localized in mitochondria, can also protect against Wallerian degeneration when overexpressed in transgenic mice (Yahata et al., 2009). Interestingly, neither NMNAT1 nor NMNAT3, but NMNAT2 is required for axon integrity (Gilley and Coleman, 2010). After axons are injured, NMNAT2 expression rapidly declines promoting axon degeneration (Araki et al., 2004; Sasaki et al., 2006; Babetto et al., 2013).

Previous work found that axonal pruning during early development was not altered in Wld^S^ mice and flies overexpressing the Wld^S^ protein (Hoopfer et al., 2006). This suggested that Wallerian degeneration only occurs after injury and not during development. However, it has become clear that early cortical development driven by spontaneous activity and critical period plasticity driven by experience are regulated by different molecular and cellular mechanisms (Hensch, 2005; Levelt and Hübener, 2012; Lohmann and Kessels, 2014). Therefore, we wanted to reassess the premise that the Wallerian degeneration signaling pathway is not involved in normal development of the brain.

To this aim, we assessed whether OD plasticity is altered in mouse lines in which Wallerian degeneration is reduced due to overexpression of cytoplasmic NMNAT overexpression. We find that in mice overexpressing NMNAT in the cytoplasm, OD plasticity is reduced during the second half of the critical period but not during the first half. Moreover, we find that cortical visual acuity in Wld^S^ is already high at a young age. At the same time, we find that OD plasticity does not cause hallmark signatures of Wallerian degeneration. Together, these results suggest that genes that regulate Wallerian degeneration also regulate developmental events in the visual cortex.

## Materials and Methods

### Animals

We made use of mice overexpressing the Wld^S^ protein, NMNAT1 (nuclear isoform) or NMNAT3 (mitochondrial isoform; Yahata et al., 2009). The Wld^S^ line was originally derived from the C57Bl/6Ola/hsd mouse line and hence, these mice were used as wild-type controls for these mice. NMNAT1 and NMNAT3 mice were maintained on a C57Bl6/j background and wild-type littermates were used as controls. Mice of either sex were used for all experiments. All experiments involving mice were approved by the institutional animal care and use committee.

### Surgical preparation

For MD, the upper and lower lids of the right eye were clipped and sutured together with two mattress sutures during isoflurane anesthesia. During the procedure the eye was rinsed with saline and after suturing lidocaine cream was applied to the closed eyelid. At the start of an imaging session, the eyes were reopened. Animals which had early opening of the eye or a damaged eye were excluded from the experiments.

### Optical imaging and visual stimulation

Optical imaging of intrinsic signal was performed as previously described (Heimel et al., 2007). Mice were anesthetized with an intraperitoneal injection of urethane (Sigma; 20% solution in saline, 2 mg/10 g bodyweight). This was immediately followed by a subcutaneous injection of atropine sulphate (AUV; 50 µg/ml in saline, 1 µg/10 g bodyweight) to reduce excretions from mucous membranes and chlorprothixene (Sigma; 2 mg/ml in saline, 80 µg/10 g bodyweight). Sometimes a supplement of urethane of ∼10% of the initial dose was necessary to obtain a sufficient amount of anesthesia. Anesthetized mice were placed on a heating pad and body temperature was monitored with a rectal probe and maintained at 36.5°C. A continuous flow of oxygen was provided close to the nose. The mouse was fixated by ear bars with conical tips and a bite rod behind the front teeth, 3 mm lower than the ear bars. The scalp was treated with xylocaine (lidocaine HCl, AUV), and part of the scalp was removed to expose the skull. The skull was cleaned using saline. Black cloth was used to prevent light from the monitor reaching the camera. Light from a tungsten-halogen lamp filtered through a KG-1 heat filter and a 700 nm (30 nm width) bandpass filter illuminated the skull. Reflecting light was caught by Adimec-1000m/D CCD camera behind a macroscope composed of two Nikkor 50mm/f1.2 lenses, focused 0.8 below the cranial surface, centred at 2.6 mm lateral and 0.5 rostral to lambda. Images, taken at 25 Hz, were down sampled and stored 1.7 Hz by an Optimal Imager 3001 system (Optical Imaging Inc).

A γ-corrected Dell UltraSharp U2312HM 23′′ full HD LCD monitor was placed at 15 cm from the mouse’ eye contralateral to the imaged hemisphere covering –15–75° horizontally and –45–45° vertically of the visual field. Background luminance was 5 cd m^−2^. For obtaining a coarse retinotopic map, the screen was divided in two by two rectangles. Square wave, 90% contrast, gratings of 0.05 cycles per degree drifting at 40° per second and changing drifting directions every 0.6 s, were intermittingly shown for 6 s every 15 s in one of the quadrants, on an equiluminant gray background, for ∼15 repetitions. For measuring OD, the same square wave gratings were shown in the superior-nasal quadrant, for 3 s per stimulation. Using automated eye shutters, vision was allowed through the left, the right or no eye (to check the completeness of the vision block) in random order (at least 40 repetitions for each condition). Acuity was measured by stimulating the contralateral eye with sinusoidal, 90% contrast, gratings of various spatial frequencies, for at least 40 repetitions. Visual stimuli were separated by periods of equiluminant gray of at least 12 s.

Image analysis was done by first subtracting signal average of the last 3 s before stimulation. To remove global slow biological fluctuations, this signal was normalized by changes occurring in a reference region outside of visual cortex. For each pixel, the response was computed as the negative of this average signal during the visual stimulation. For retinotopic mapping, each pixel received a color corresponding to the quadrant to which the signal was highest. Using this map, the area corresponding to the superior-nasal quadrant was manually selected as ROI for the OD and acuity measurements. The response in these tests was taken as the mean response over all pixels in this ROI. The imaged OD index (iODI) was defined as (contra-response – ipsi-response)/(contra-response + ipsi-response). The acuity was defined by the intercept with the spatial frequency axis after fitting the spatial frequency tuning curve with a downward sloping threshold linear function.

### Immunohistochemistry

Age matched mice were anesthetized with 0.1 ml/g body weight Nembutal (Janssen) and perfused with 4% paraformaldehyde (PFA) in PBS (∼80 ml per mouse) and postfixed for 2–3 h. Coronal sections of 50 µm were made by using a vibratome (Leica VT1000S). Antibodies used were against synaptotagmin-2 (rabbit, 1:1000, a kind gift from Dr. T. Südhof) followed by Alexa Fluor 568-conjugated goat anti-rabbit antibody (1:000, A11011, Invitrogen) or the F4/80 marker for activated microglia (mouse, 1:200, MCA497, Bio-Rad) followed by Alexa Fluor 568-conjugated goat anti-mouse antibody (1:500, A11004, Invitrogen). All antibodies were previously tested in mice for the application we used them for (more information can be found in the references or on the websites of the suppliers). Free-floating sections were briefly washed in PBS followed by 1 h blocking in PBS containing 5% normal goat serum and 0.1% Triton X-100. Primary antibody incubation was performed overnight at 4°C in fresh blocking solution. Next, the sections were washed three times for 10 min in PBS with 0.1% Tween 20 (PBST) followed by secondary antibody incubation in fresh blocking solution for 90 min at room temperature (RT). After washing three times for 10 min in PBST, the sections were mounted on glass slides using Mowiol (Calbiochem/MerckMillipore) and glass covered for imaging.

### Confocal microscopy and data analysis

For quantification of synaptotagmin-2 puncta number, fluorescent puncta were analyzed using a non-commercially available macro for Image-Pro PLUS (v6.3). Up to six puncta rings per image obtained from V1 sections were manually encircled after which a mask was created on the cell. A 2-µm-wide ring was calculated around the mask and all puncta in the ring were considered to belong to the cell and were counted and measured. Signals not reaching size and fluorescent threshold levels were omitted. Pixel intensity for the signal within masks was considered background and subtracted from the intensity values in the puncta. Puncta numbers were analyzed per image. For quantification of the number of activated microglia, the number of microglia cells were manually counted in images obtained from V1 sections. An area of 387.5 × 387.5 µm was counted. Only cells of which the soma was visible were included.

### qPCR

RNA was isolated from tissue containing V1 from both control and Wld^S^ mice using the *mir*Vana miRNA isolation kit (Invitrogen). Total RNA (200 ng) was DNase I treated and used as a template to generate cDNA following the manufacturer's instructions (QuantiTect Reverse Transcription kit; QIAGEN) with a blend of oligo-dT and random primers. The reverse transcriptase reaction was incubated at 42°C for 30 min and terminated at 95°C for 3 min. The resulting cDNA was diluted 1:20 and served as a template in real-time qPCR assays (SYBR-Green PCR Master Mix; Applied Biosystems). Primers were generated for *GAD65* and tested for efficiency. The determined transcript levels of these target genes were normalized against the levels of *GAPDH* determined in the same sample to control for variability in the amount and quality of the RNA and the efficiency of the cDNA reaction.

### Slice electrophysiology

Mice were anesthetized using isoflurane and then decapitated. Brains were quickly removed and kept at 0°C in carbogenated (95% O_2_/5% CO_2_) modified ACSF containing choline chloride (110 mM choline chloride, 7 mM MgCl_2_, 0.5 mM CaCl_2_, 2.5 mM KCl, 11.6 mM Na-ascorbate, 3.10 mM Na-pyruvate, 1.25 mM NaH_2_PO_4_, 25 mM D-glucose, and 25 mM NaHCO_3_), to prevent axon potentials in the brain during stressful conditions; 330-µm-thick coronal slices containing the visual cortex were cut on a vibratome (Microm HM650V; Thermo Scientific) while keeping the slices in carbogenated modified ACSF (125 mM NaCl, 3 mM KCl, 2 mM MgSO_4_, 2 mM CaCl_2_, 10 mM glucose, 1.20 mM NaH_2_PO_4_ and 26 mM NaHCO_3_) at 0°C. After slicing, all slices were kept in ACFS at 35°C for 30–45 min for recovery, while continuously bubbled with carbogen. Next, slices were kept in continuously carbogenated ACSF at RT until use (1–6 h after slicing). To perform electrophysiological experiments, slices were moved to a chamber with continuous inflow and outflow of carbogenated ACSF at a rate of 1–2 ml/min at RT. For all experiments, a layer 2/3 pyramidal neuron in the visual cortex was patched. A glass pipette with a resistance between 3 and 6 MΩ was filled with intracellular solution containing 1mg/ml biocytin for *post hoc* staining of the patched cell. After obtaining a gigaOhm seal, whole-cell patch clamp recordings were performed using Axopatch 1D (Molecular Devices). When the cell was patched, several currents were injected to see whether a cell was healthy and whether it showed a firing pattern typical for a pyramidal neuron. Before recording miniature EPSCs (mEPSCs), the bath solution was replaced with ACSF containing 1 µM TTX to block all voltage dependent sodium currents and 20 µM gabazine to block all GABA_A_ receptors. For all experiments, cells were clamped at –70 mV, and mEPSCs were measured during 5 min. Mini Analysis (Synaptosoft Inc.) was used for analyzing mEPSCs. Recordings were included when the seal resistance >1 GΩ, the series resistance was smaller than 20 MΩ, the whole cell capacitance was smaller than 150 pF, the resting potential was more negative than –60 mV, and the RMS noise was <2.5 pA (threshold cutoff in MiniAnalysis was set at 6, which is 2–2.5 times the value of the RMS noise), before and after recording.

### Western blot analysis

V1 from Wld^S^ and control mice and the binocular part of V1 from control mice with or without MD were collected and homogenized in lysis buffer (LB) containing 150 mM sodium chloride, 1% Triton X-100, 50 mM Tris, pH 8, and a protease inhibitor cocktail (cOmplete Mini EDTA-Free, Roche), using an electric homogenizer (IKA). Proteins were purified by centrifugation (1000 × *g*), and the supernatant was collected. Protein content was measured by comparing with a bovine serum albumin standard using a bicinchoninic acid (BCA) reaction kit (Thermo Fisher Scientific/Pierce), and the optical density of the reagent was measured with an iEMS Reader MF (Labsystems/Thermo Scientific). Western blotting was performed using the NuPAGE Novex Bis-Tris pre-cast gel kit with a 4–12% gradient or the NuPAGE Novex Tris-acetate pre-cast gel kit with a 3–8% gradient, in Invitrogen gel containers (Invitrogen). Gels were loaded with 60-µg protein and run for 1 h at 200 V (Bis-Tris gel) or 150 V (Tris-acetate gel). After separation, gels were transferred at 14 V overnight to PVDF Immobilon-FL transfer membranes (Millipore). Protein membrane blots were stained with antibodies against synapsin 1 (rabbit, 1:250, ab64581), synaptotagmin-2 (mouse, 1:250, znp-1, ZIRC), NMNAT2 (mouse, 1:50, B_10_, Santa Cruz Biotechnology), MYCBP2 (rabbit, 1:500, ab86078, Abcam), and GAPDH (mouse, 1:1000,MAB374, Merck) and analyzed using infrared secondary antibodies (LI-COR Biosciences) and the Odyssey Infrared Imaging System (LI-COR). Imaged bands were measured using the Odyssey application software and corrected for background intensities from adjacent non-labeled lanes.

### Statistics

We determined that the imaged OD index values were normally distributed using the Shapiro–Wilk test. For testing differences in OD plasticity between transgenic and control mice, we computed whether there was a significant interaction in a two-way ANOVA. For graphical representation of significant differences between groups, we presented the results of *post hoc* Tukey–Kramer tests. Because puncta number and density, mEPSCs, Western blotting data, and *GAD65* mRNA levels were normally distributed (Shapiro–Wilk test), we used *t* test when two independent groups were compared.

## Results

### Reduced OD plasticity in Wld^S^ mice

**Figure 1.**
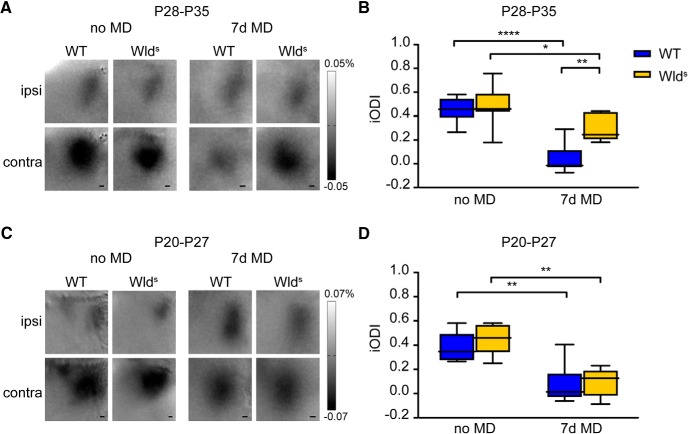
Wld^S^ mutant mice have reduced OD plasticity during the critical period. ***A***, Transcranial images of change in light reflection in V1, in response to individual eye stimulation in non-deprived (no MD) and 7 D-deprived (7d MD) wild-type (WT) and Wld^S^ mice, during the peak of the critical period at P28–P35. ***B***, iODI shows that 7d MD induces a larger OD shift in WT mice than in Wld^S^ mutant mice (interaction genotype/OD-shift: two-way ANOVA, *p* = 0.0029, *post hoc* Tukey’s, WT vs WT MD, *p* < 0.0001, Wld^S^ vs Wld^S^ MD, *p* = 0.0114, WT MD vs Wld^S^ MD, *p* = 0.0034, WT: *n* = 12 mice, Wld^S^: *n* = 9 mice, WT MD: *n* = 8 mice, Wld^S^ MD: *n* = 7 mice). ***C***, Transcranial images of change in light reflection in V1, in response to individual eye stimulation in no MD and 7d MD WT and Wld^S^ mice, before the peak of the critical period. ***D***, Imaged ODIs show that earlier in development MD induces an OD shift in both WT and Wld^S^ mice, with no significant difference between the genotypes (interaction genotype/OD-shift: two-way ANOVA, *p* = 0.4018; interaction treatment/OD-shift: *p* < 0.0001, *post hoc* Tukey’s, WT vs WT MD, *p* = 0.0022, Wld^S^ vs Wld^S^ MD, *p* = 0.0021. WT: *n* = 8 mice, Wld^S^: *n* = 5 mice, WT MD: *n* = 6 mice, Wld^S^ MD: *n* = 5 mice). Values shown as median (solid line), ±1.5 interquartile range (box) and minimal and maximal values (whiskers). Scale bars, 200 µm; *p < 0.05, **p < 0.01, ****p < 0.0001.

We first set out to investigate whether the Wld^S^ mutation affects OD plasticity. To this end, we used optical imaging of intrinsic signal to determine the OD in V1 of Wld^S^ mice and control C57Bl/6Ola/hsd mice that were either MD for 7 d during the peak of the critical period [postnatal day (P)28–P35] or reared normally. We found that 7 d of MD caused a stronger shift in OD in wild-type mice than in Wld^S^ mice (Fig. 1*A*,*B*). Thus, the Wld^S^ mutation interferes with efficient critical period plasticity in V1.

### Unaffected plasticity and higher visual acuity in V1 of young Wld^S^ mice

Axon degeneration involves the decreasing availability of NMNAT2 (Gilley and Coleman, 2010), a process that is enhanced by the Phr1 E3 ubiquitin ligase MYCBP2 (Babetto et al., 2013). Interestingly, MYCBP2 was found to down-regulate in V1 between P30 and P46 (Dahlhaus et al., 2011). It is thus possible that this leads to a gradual increase of axon stability contributing to declining plasticity. We therefore wanted to test whether the reduced plasticity observed in P28–P35 Wld^S^ mice was not a plasticity deficit per se, but a selective decline of plasticity during the later phase of the critical period. To this aim, we assessed whether at an earlier age, Wld^S^ mice did show OD plasticity. We again used optical imaging of intrinsic signal to determine the OD in Wld^S^ mice and control C57Bl/6Ola/hsd mice that were reared normally, or MD for 7 d, but now starting one week earlier (P20–P27). We found that 7 d of MD starting at P20 induced a full OD shift in Wld^S^ mice, similar to what we observed in C57Bl/6Ola/hsd mice (Fig. 1*C*,*D*). Therefore, we conclude that in Wld^S^ mice, OD plasticity is not deficient as such, but is decreased during the second half of the critical period.

**Figure 2.**
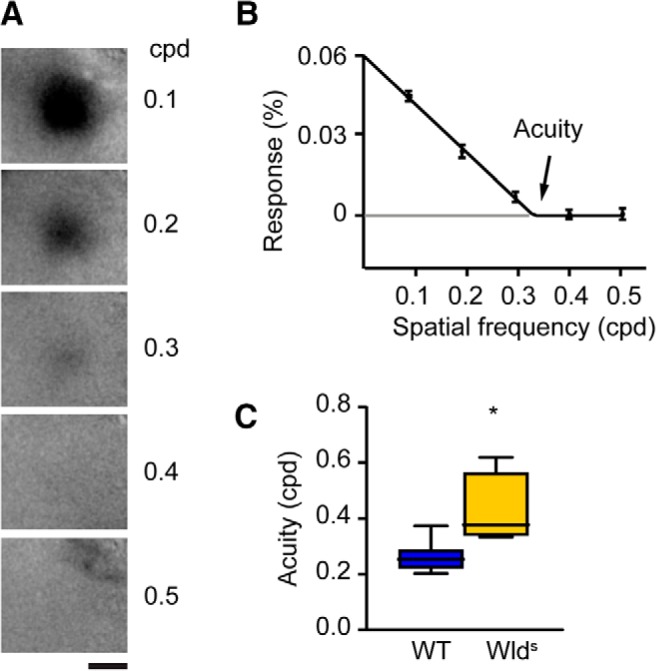
Early increase of visual acuity in Wld^S^ mice. ***A***, Transcranial images of light reflection in V1, in response to visual stimulation using sinusoidal gratings with different spatial frequencies in cycles per degree (cpd). Scale bar, 1 mm. ***B***, Example of cortical responses and the inferred acuity of a wild-type (WT) mouse. ***C***, Acuity of V1 responses is significantly increased in Wld^S^ mutant mice compared to WT mice at P25 (*t* test, *p* = 0.015, WT: *n* = 7, Wld^S^: *n* = 4).

This finding suggested that other functional changes that normally occur around the end of the critical period may also take place at a younger age in Wld^S^ mice. One such change is the increase in cortical visual acuity from ∼0.25 cpd at P25 to over 0.5 cpd at P35 (Gordon and Stryker, 1996; Huang et al., 1999; Gianfranceschi et al., 2003; Heimel et al., 2007). We determined acuity in both Wld^S^ and control C57Bl/6Ola/hsd mice at P25, by making use of optical imaging of intrinsic signal (Fig. 2*A*,*B*). It was previously shown that at this age, visual acuity is still low in wild-type mice, but nearly at adult levels in mice overexpressing BDNF causing premature cortical development (Huang et al., 1999). Similarly, we observed that at P25, visual acuity in Wld^S^ mice was significantly higher than in control mice (0.4 vs 0.25 cpd; Fig. 2*C*). Thus, acuity in V1 reaches high levels at a younger age in Wld^S^ mice than in control mice.

### No evidence for altered inhibitory or excitatory synapse development in Wld^S^ mice

**Figure 3.**
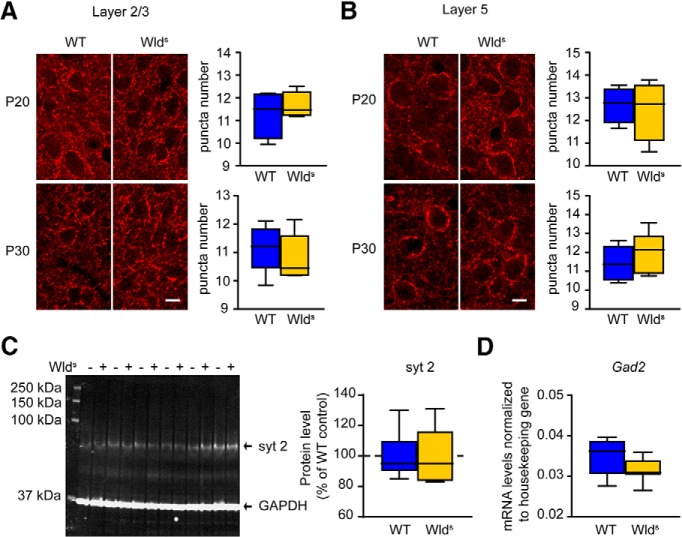
Normal development of inhibitory innervation in Wld^S^ mice. ***A***, Representative images showing synaptotagmin-2 (syt 2)-positive puncta forming rings around cell bodies in cortical layers 2/3 in wild-type (WT) and Wld^S^ mice at P20 and P30. Number of syt 2-expressing boutons forming puncta rings in layers 2/3 are unchanged in Wld^S^ mice compared to WT controls at P20 (*t* test, *p* = 0577, *n* = 4 for both genotypes) or P30 (*p* = 0521, *n* = 5 for both genotypes). ***B***, Representative images showing syt 2-positive puncta forming rings around cell bodies in cortical layer 5 in WT and Wld^S^ mice at P20 and P30. Number of syt 2-expressing boutons forming puncta rings in layer 5 are unchanged in Wld^S^ mice compared to WT controls at P20 (*t* test, *p* = 0785, *n* = 4 for both genotypes), or P30 (*p* = 0461, *n* = 5 for both genotypes). ***C***, Representative examples of Western blot analysis of V1 and quantification of expression levels, normalized to those of WT control mice. Expression levels for syt 2 are unaltered at P30 (*t* test, *p* = 0.9869, *n* = 6 for both genotypes). ***D***, *GAD65* mRNA expression levels relative to housekeeping gene is unchanged at P30 (*p* = 0.089, WT: *n* = 8, Wld^S^: *n* = 7). Values shown as median (solid line), ±1.5 interquartile range (box) and minimal and maximal values (whiskers). Scale bars, 10 µm.

In BDNF-overexpressing mice, an early decline of OD plasticity and accelerated development of visual acuity was associated with accelerated development of the GABAergic inhibitory system (Hanover et al., 1999; Huang et al., 1999). One of the key players in the regulation of critical periods are the parvalbumin-expressing (PV+) basket cells (Chattopadhyaya, 2004; Fagiolini, 2004; Hensch, 2005; Kuhlman et al., 2013). The axon terminals of these PV+ neurons form presynaptic boutons on the soma and proximal dendrites of their target neuron and the development of the puncta-rings that they form coincides with the critical period (del Río et al., 1994). We therefore set out to quantify the difference in perisomatic inhibition from PV+ interneurons onto pyramidal neurons in layer 2/3 and layer 5 of V1 in Wld^S^ mice and control C57Bl/6Ola/hsd mice, by staining for synaptotagmin-2, a protein selectively expressed by PV+ boutons (Sommeijer and Levelt, 2012). Using immunohistochemistry and confocal microscopy, we assessed the density of synaptotagmin-2 puncta at different developmental ages, P20 and P30. However, we did not detect a difference in density of perisomatic synaptotagmin-2 puncta in Wld^S^ mice compared to wild-type mice (Fig. 3*A*,*B*). Additionally, we studied whether there is higher expression of synaptotagmin-2. Western blot analysis of V1 from P30 Wld^S^ and control C57Bl/6Ola/hsd mice revealed no difference in synaptotagmin-2 levels (Fig. 3*C*). These findings suggest that inhibitory innervation through PV+ interneurons is not responsible for the early functional changes in V1 of Wld^S^ mice. To further investigate this, we studied the levels of the glutamic acid decarboxylase GAD65, an enzyme involved in GABA synthesis. We used quantitative PCR to determine levels of *Gad2* mRNA (encoding GAD65) relative to housekeeping genes in V1 from P30 mice but found no difference between Wld^S^ and wild-type control mice (Fig. 3*D*). Thus, our findings do not support the notion that development of GABAergic innervation is accelerated in Wld^S^ mice.

**Figure 4.**
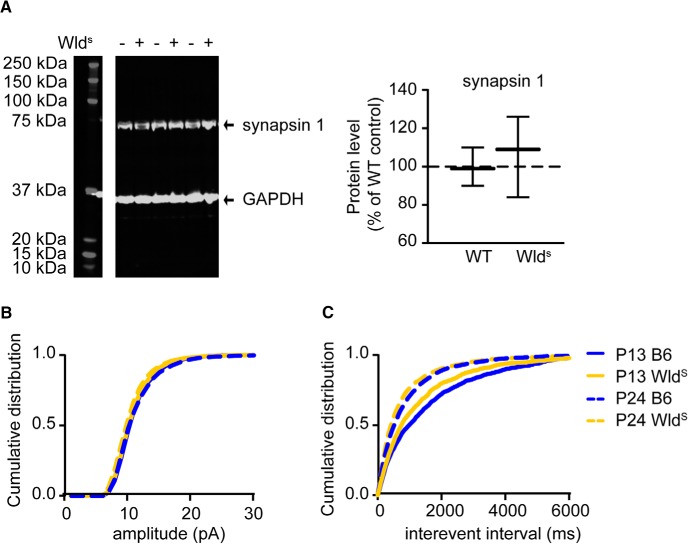
Unaltererd cortical excitation in Wld^S^ mutant mice. ***A***, Representative examples of Western blot analysis of V1 and quantification of expression levels, normalized to those of wild-type (WT) control mice. Expression levels for synapsin 1 are unaltered at P20 (*t* test, *p* = 0.5091, *n* = 6 for both genotypes). Values shown as median (solid line), ±1.5 interquartile range (box) and minimal and maximal values (whiskers). ***B***, ***C***, Cumulative distributions of the amplitude (***B***) or interevent interval (***C***) of mEPSC in Wld^S^ (yellow) or WT (blue) mice at P13–P14 (solid line) or P24–P25 (dotted line). Excitatory input to pyramidal neurons shows no significant change in amplitude between Wld^S^ and WT control mice at P13–P14 (*t* test, *p* = 0.55, WT: *n* = 10, Wld^S^: *n* = 5) or P24–P25 (*p* = 0.59, WT: *n* = 9, Wld^S^: *n* = 13). Frequencies of mEPSCs in Wld^S^ mice and WT controls also show no significant difference between the two genotypes (P13–P14: *p* = 0.84, P24–P25: *p* = 0.23, n = same is as in ***A***).

Previous studies have shown that also excitatory synapse maturation affects critical period onset and closure (Bochner et al., 2014; Stephany et al., 2014; Huang et al., 2015; Jenks et al., 2017). We therefore studied whether excitatory synapses developed differently in Wld^S^ mice. To determine this, we first quantified levels of the presynaptic protein synapsin 1 in dissected V1 from Wld^S^ and control C57Bl/6Ola/hsd mice at p20. We found no differences in synapsin 1 levels between these mice (Fig. 4*A*). To also have a functional readout of excitatory synapse development, we measured the amplitude and interevent intervals of mEPSCs in pyramidal neurons in layers 2/3 of V1 in Wld^S^ and wild-type mice of P13–P14 and P24–P25. We found no differences in mEPSC amplitudes or interevent intervals between Wld^S^ and wild-type mice (Fig. 4*B*,*C*). Thus, more rapid excitatory synapse development also does not seem to explain the reduced OD plasticity in P28–P35 Wld^S^ mice.

### NMNAT3, but not NMNAT1, overexpression reduces OD plasticity

In Wld^S^ mice, a UBE4b-NMNAT1 fusion protein is overexpressed. The effect that this protein has on slowing down Wallerian degeneration depends on its presence in axons (Conforti et al., 2007). However, the Wld^S^ fusion protein is also present in the nucleus. Endogenous NMNAT1 is a nuclear NAD+ synthase and is involved in transcriptional regulation (Pollak et al., 2007; Chang et al., 2010). It is thus conceivable that the plasticity changes we observe in Wld^S^ mice are not caused through the signaling pathway that regulates Wallerian degeneration, but through nuclear NMNAT activity.

**Figure 5.**
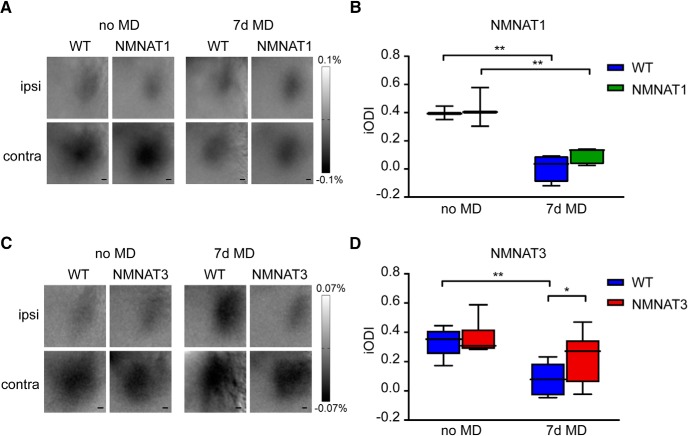
NMNAT3, but not NMNAT1, overexpression reduces OD plasticity. ***A***, Transcranial images of change in light reflection in V1, in response to individual eye stimulation in non-deprived (no MD) and 7 D-deprived (7d MD) wild-type (WT) and NMNAT1 mice. ***B***, iODI shows that 7d MD during the peak of the critical period induces an OD shift both in WT mice and in NMNAT1 mice (interaction genotype/OD-shift: two-way ANOVA, *p* = 0.235; interaction treatment/OD-shift: *p* < 0.0001, *post hoc* Tukey’s, WT vs WT MD, *p* = 0.0033, NMNAT1 vs NMNAT1 MD, *p* = 0.0015. WT: *n* = 3 mice, NMNAT1: *n* = 3, WT MD: *n* = 4, NMNAT1 MD: *n* = 5). ***C***, Transcranial images of change in light reflection in V1, in response to individual eye stimulation in no MD and 7d MD WT and NMNAT3 mice. ***D***, Imaged ODIs show that 7d MD during the peak of the critical period, induces a larger OD shift in WT mice than in NMNAT3 mice (interaction genotype/OD-shift: two-way ANOVA, *p* = 0.0475; interaction treatment/OD-shift: *p* < 0.0001, *post hoc* Tukey’s, WT vs WT MD, *p* = 0.001, WT MD vs NMNAT3 MD, *p* = 0.0283, NMNAT3 vs NMNAT3 MD, *p* = 0.09. WT: *n* = 7, NMNAT3: *n* = 8, WT MD: *n* = 11, Wld^S^ MD: *n* = 16). Values shown as median (solid line), ±1.5 interquartile range (box) and minimal and maximal values (whiskers); *p < 0.05, **p < 0.01.

To test this possibility, we examined whether OD plasticity was reduced in a transgenic mouse line overexpressing nuclear NMNAT1 (Yahata et al., 2009). Again, we used optical imaging of intrinsic signal to determine OD in NMNAT1 transgenic mice and wild-type littermates that were either reared normally, or monocular deprived for 7 d (P28–P35). We found that OD plasticity occurred normally in NMNAT1-overexpressing mice (Fig. 5*A*,*B*), suggesting that the Wld^S^ mutation does not cause accelerated cortical development by directly altering transcriptional control.

To test the alternative hypothesis that increased cytoplasmic NMNAT activity is responsible for decreased OD plasticity toward the end of the critical period, we made use of mice overexpressing NMNAT3. Previous work has shown that in contrast to NMNAT1, overexpression of NMNAT3 slows down Wallerian degeneration, possibly by substituting for cytoplasmic NMNAT2 when it is down-regulated (Avery et al., 2009; Yahata et al., 2009). We found that in NMNAT3-overexpressing mice, OD plasticity after 7 d of MD was significantly reduced (Fig. 5*C*,*D*), mimicking the phenotype of Wld^S^ mice. This indicates that the effects observed in Wld^S^ mice are caused by cytoplasmic effects of the UBE4b-NMNAT1 fusion protein rather than by its nuclear activity. These results suggest that the reduction of OD plasticity in Wld^S^ mice during the last week of the critical period probably involves a signaling cascade that involves genes that regulate Wallerian degeneration.

### MD does not affect expression of Wallerian degeneration-related proteins

**Figure 6.**
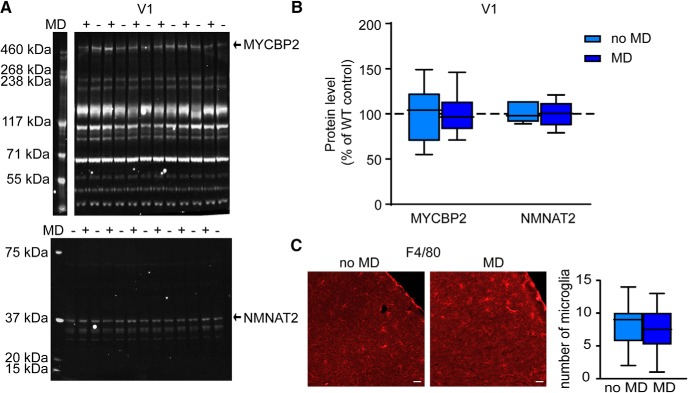
MD does not affect expression of Wallerian degeneration-related proteins. ***A***, Representative examples of Western blot analysis of V1 homogenates of wild-type (WT) mice at P37 with (+) or without (–) 7 d MD for MYCBP2 and NMNAT2 expression. ***B***, Quantification of expression levels, normalized to those of non-deprived WT control mice. Expression levels for both MYCBP2 and NMNAT2 are unaltered in V1 after MD (*t* test, MYCBP2: *p* = 0.992, both conditions: *n* = 6; NMNAT2: *p* = 0.985, no MD: *n* = 7, MD: *n* = 6). ***C***, Examples and quantification of F4/80 stainings for activated microglia in WT control mice with or without 7 d MD. The number of activated microglia per 387.5 × 387.5 µm area of V1 is not significantly different with or without 7 d MD (*t* test, *p* = 0.821, no MD: *n* = 4 mice; MD: *n* = 3 mice). Values shown as median (solid line), ±1.5 interquartile range (box) and minimal and maximal values (whiskers). Scale bars, 50 µm.

So far, our results show that the signaling pathway that regulates Wallerian degeneration also causes the decline of OD plasticity to occur prematurely in V1. This could imply that OD plasticity actually involves Wallerian degeneration. Alternatively, Wallerian degeneration and cellular events underlying OD plasticity, such as axon growth and retraction and synapse turnover, use partially overlapping signaling cascades. To differentiate between these possibilities, we looked for direct signs of Wallerian degeneration during the induction of OD plasticity, such as the presence of activated microglia, or acute regulation of MYCBP2 or NMNAT2 protein levels (Babetto et al., 2013). To determine changes in the levels of MYCBP2 and NMNAT2 after MD, we performed quantitative Western blot analysis on V1 dissected from the contralateral hemisphere of mice that were MD for 7 d and undeprived littermates. No differences in NMNAT2 or MYCBP2 levels were found between tissue from non-deprived and deprived mice (Fig. 6*A*,*B*). This suggests that OD plasticity does not involve Wallerian degeneration initiated by MYCBP2/NMNAT2 regulation. We also assessed whether OD plasticity resulted in the presence of activated microglia, a hallmark of Wallerian degeneration. We performed immunohistochemistry with the activated microglia-specific antibody F4/80 (Castaño et al., 1996) on sections of V1 of C57Bl/6Ola/hsd mice that had been subjected to 7 d of MD and undeprived littermates. Assessment of F4/80 stained V1 sections did not reveal a significant increase in active microglia between non-deprived and deprived mice (Fig. 6*C*,*D*). Thus, we did not find evidence for Wallerian degeneration occurring during OD plasticity, despite the finding that both events are regulated by NMNAT proteins.

## Discussion

In this study, we show for the first time that genes known for regulating Wallerian degeneration also control experience-dependent plasticity during development. We demonstrate that compared to control mice, OD plasticity is lower toward the end of the critical period in Wld^S^ mice and visual acuity increases at an earlier age. Interestingly, we did not find any signs of Wallerian degeneration during OD plasticity, suggesting that these processes share a common signaling pathway but do not necessarily involve the same cellular mechanisms of axon degeneration.

The Wld^S^ UBE4b-NMNAT1 fusion protein slows down Wallerian degeneration by mislocalizing NMNAT1 to the cytoplasm (Conforti et al., 2007; Sasaki et al., 2016), where it prevents SARM1-dependent NAD+ depletion that drives axonal disintegration (Sasaki et al., 2016). Endogenous NMNAT1 is expressed in the nucleus however, where it controls NAD+ synthesis and modulates gene transcription (Chang et al., 2010). Our experiments confirm that increased cytoplasmic NMNAT levels also interfere with OD plasticity: in mice overexpressing nuclear NMNAT1, OD plasticity was unaffected while it was reduced in mice overexpressing NMNAT3 which localizes to mitochondria and also reduces Wallerian degeneration (Avery et al., 2009; Yahata et al., 2009). Whether increased cytoplasmic NMNAT reduces OD plasticity through preventing SARM1-dependent NAD+ depletion or through a different signaling cascade remains to be tested.

An earlier study had revealed that axon pruning during development is not altered in Wld^S^ mice or flies expressing the Wld^S^ protein (Hoopfer et al., 2006). This implies that the Wallerian degeneration pathway is only active during pathologic axon retraction and does not regulate physiologic axon retraction during development. The fact that OD plasticity is affected in Wld^S^ mice and mice overexpressing NMNAT3 shows that the situation is more complex and that during a later phase of cortical development the Wallerian degeneration signaling pathway does have a role in a physiologic developmental process. We were not able to determine whether the reduced OD plasticity at the end of the critical period was due to a deficit in axon pruning. We did not find any direct evidence for Wallerian degeneration during OD plasticity in V1, such as altered NMNAT2 or MYCBP2 expression (Gilley and Coleman, 2010) or obvious changes in microglia activation. However, it is possible that OD plasticity does not involve the synchronous retraction of a sufficiently large population of cortical axons to cause detectable changes in these markers. Alternatively, NMNAT proteins may not be involved in acute axonal destabilization during OD plasticity, but regulate cortical plasticity by gradually increasing overall axon or synapse stability in the maturing visual cortex.

We found no evidence for activation of microglia after MD. This is in line with a previous study that showed that microglia cells contributed to OD plasticity but did not become activated (Tremblay et al., 2010). However, in this study more subtle changes in microglia morphology and dynamics were observed by *in vivo* two-photon microscopy after brief MD that were essential for effective OD plasticity (Tremblay et al., 2010). Thus, in contrast to the full microglia activation and degeneration of entire axons in Wallerian degeneration after cortical damage, OD plasticity seems to result in a more subtle process involving partial activation of microglia and limited retraction of axonal branches.

An early decline of OD plasticity has been observed previously in mice overexpressing BDNF (Hanover et al., 1999; Huang et al., 1999). In these mice, the increase in visual acuity that usually occurs between P25 and P35 also happened at an earlier age, as did the maturation of inhibitory innervation. It is thus possible that also in Wld^S^ mice, the visual cortex matures faster. We did indeed observe that cortical visual acuity was higher at P25 in Wld^S^ mice than in control mice. However, we did not observe a more rapid development of cortical inhibitory innervation in Wld^S^ mice. We also did not find evidence for accelerated excitatory synapse development. Possibly, more subtle developmental alterations of the visual system underlie the rapid increase in visual acuity in Wld^S^ mice, such as changes in cortical AMPA/NMDA receptor ratios (Saiepour et al., 2017) or thalamic wiring (Stephany et al., 2018). Future studies will have to reveal whether earlier events in visual cortical development, such as transitions in spontaneous activity patterns (Siegel et al., 2012), binocular orientation matching (Wang et al., 2010) or critical period onset (Hensch et al., 1998), also occur at an earlier age in Wld^S^ mice.

In conclusion, this study provides the first evidence that genes in the signaling pathway regulating Wallerian degeneration are also involved in the control of experience-dependent plasticity during normal cortical development. This knowledge may help to reveal the mechanisms involved in critical period closure during cortical development and advance the discovery of novel drug targets for enhancing brain plasticity for therapeutic purposes.

## Synthesis

Reviewing Editor: Christophe Bernard, INSERM & Institut de Neurosciences des Systèmes Decisions are customarily a result of the Reviewing Editor and the peer reviewers coming together and discussing their recommendations until a consensus is reached. When revisions are invited, a fact-based synthesis statement explaining their decision and outlining what is needed to prepare a revision will be listed below. The following reviewer(s) agreed to reveal their identity: Tobias Stork.

The authors must be commended for improving the clarity of the manuscript.

There are minor issues that can be easily addressed.

1) The authors agree that as is, the experiments performed would not be able to determine whether the critical period closes prematurely. However, the rationale presented on line 228-230 still implies that their experiments will be able to answer this question.

2) Line 236 OD plasticity is decreased at an early age - change to late age

3) As written, “acuity in V1 reaches high levels at a younger age in WldS mice than in control mice” (line 246) implies that WT mice will reach the same ‘high’ level of acuity as WldS mice. Stating that the reported range of adult WT acuity is XXX cpd should clarify the significance of the result. Otherwise, the conclusion would be more accurate if stated: “acuity is higher in WldS than in control mice at age P25”.

4) The authors decided not to analyze sarm mutant mice, which is fine. We would suggest to include a comment in the discussion to this effect.

5) The section “optical imaging and visual stimulation” in the methods and materials part seems quite inadequate and the actual imaging part is basically omitted (e.g. what camera was used etc.). Either a more detailed description should be provided or an adequate paper should be cited here.

## References

[B1] Antonini A, Fagiolini M, Stryker MP (1999) Anatomical correlates of functional plasticity in mouse visual cortex. J Neurosci 19:4388–406. 10341241 10.1523/JNEUROSCI.19-11-04388.1999 PMC2452998

[B2] Araki T, Sasaki Y, Milbrandt J (2004) Increased nuclear NAD biosynthesis and SIRT1 activation prevent axonal degeneration. Science 305:1010–1013. 10.1126/science.1098014 15310905

[B3] Avery MA, Sheehan AE, Kerr KS, Wang J, Freeman MR (2009) Wld S requires Nmnat1 enzymatic activity and N16- VCP interactions to suppress Wallerian degeneration. J Cell Biol 184:501–513. 10.1083/jcb.200808042 19237597 PMC2654119

[B4] Babetto E, Beirowski B, Russler E, Milbrandt J, DiAntonio A (2013) The Phr1 ubiquitin ligase promotes injury-induced axon self-destruction. Cell Rep 3:1422–1429. 10.1016/j.celrep.2013.04.013 23665224 PMC3671584

[B5] Bear MF, Singer W (1986) Modulation of visual cortical plasticity by acetylcholine and noradrenaline. Nature 320:172–176. 10.1038/320172a0 3005879

[B6] Bochner DN, Sapp RW, Adelson JD, Zhang S, Lee H, Djurisic M, Syken J, Dan Y, Shatz CJ (2014) Blocking PirB up-regulates spines and functional synapses to unlock visual cortical plasticity and facilitate recovery from amblyopia. Sci Transl Med 6:258ra140. 10.1126/scitranslmed.3010157 25320232 PMC4476552

[B7] Castaño A, Lawson LJ, Fearn S, Perry VH (1996) Activation and proliferation of murine microglia are insensitive to glucocorticoids in Wallerian degeneration. Eur J Neurosci 8:581–588. 8963450 10.1111/j.1460-9568.1996.tb01243.x

[B8] Chang J, Zhang B, Heath H, Galjart N, Wang X, Milbrandt J (2010) Nicotinamide adenine dinucleotide (NAD)-regulated DNA methylation alters CCCTC-binding factor (CTCF)/cohesin binding and transcription at the BDNF locus. Proc Natl Acad Sci USA 107:21836–21841. 10.1073/pnas.1002130107 21106760 PMC3003122

[B9] Chattopadhyaya B, Di Cristo G, Higashiyama H, Knott GW, Kuhlman SJ, Welker E, Huang ZJ (2004) Experience and activity-dependent maturation of perisomatic GABAergic innervation in primary visual cortex during a postnatal critical period. J Neurosci 24:9598–9611. 10.1523/JNEUROSCI.1851-04.2004 15509747 PMC6730138

[B10] Coleman MP, Freeman MR (2010) Wallerian degeneration, wld(s), and nmnat. Annu Rev Neurosci 33:245–267. 10.1146/annurev-neuro-060909-153248 20345246 PMC5223592

[B11] Coleman MP, Conforti L, Buckmaster EA, Tarlton A, Ewing RM, Brown MC, Lyon MF, Perry VH (1998) An 85-kb tandem triplication in the slow Wallerian degeneration (Wlds) mouse. Proc Natl Acad Sci USA 95:9985–9990. 9707587 10.1073/pnas.95.17.9985 PMC21448

[B12] Conforti L, Tarlton A, Mack TG, Mi W, Buckmaster EA, Wagner D, Perry VH, Coleman MP (2000) A Ufd2/D4Cole1e chimeric protein and overexpression of Rbp7 in the slow Wallerian degeneration (WldS) mouse. Proc Natl Acad Sci USA 97:11377–11382. 10.1073/pnas.97.21.11377 11027338 PMC17208

[B13] Conforti L, Fang G, Beirowski B, Wang MS, Sorci L, Asress S, Adalbert R, Silva a, Bridge K, Huang XP, Magni G, Glass JD, Coleman MP (2007) NAD(+) and axon degeneration revisited: Nmnat1 cannot substitute for Wld(S) to delay Wallerian degeneration. Cell Death Differ 14:116–127. 10.1038/sj.cdd.4401944 16645633

[B14] Conforti L, Wilbrey A, Morreale G, Janeckova L, Beirowski B, Adalbert R, Mazzola F, Di Stefano M, Hartley R, Babetto E, Smith T, Gilley J, Billington RA, Genazzani AA, Ribchester RR, Magni G, Coleman M (2009) Wld S protein requires Nmnat activity and a short N-terminal sequence to protect axons in mice. J Cell Biol 184:491–500. 10.1083/jcb.200807175 19237596 PMC2654131

[B15] Dahlhaus M, Wan Li K, van der Schors RC, Saiepour MH, van Nierop P, Heimel JA, Hermans JM, Loos M, Smit AB, Levelt CN (2011) The synaptic proteome during development and plasticity of the mouse visual cortex. Mol Cell Proteomics 10:M110.005413. 10.1074/mcp.M110.005413 21398567 PMC3098591

[B16] del Río JA, de Lecea L, Ferrer I, Soriano E (1994) The development of parvalbumin-immunoreactivity in the neocortex of the mouse. Science 5:247–259. 10.1016/0165-3806(94)90311-5 7813046

[B17] Fagiolini M, Fritschy J-M, Löw K, Möhler H, Rudolph U, Hensch TK (2004) Specific GABAA circuits for visual cortical plasticity. Science 303:1681–1683. 10.1126/science.1091032 15017002

[B18] Geden MJ, Deshmukh M (2016) Axon degeneration: context defines distinct pathways. Curr Opin Neurobiol 39:108–115. 10.1016/j.conb.2016.05.002 27197022 PMC4987202

[B19] Gerdts J, Summers DW, Milbrandt J, DiAntonio A (2016) Axon self-destruction: new links among SARM1, MAPKs, and NAD+ metabolism. Neuron 89:449–460. 10.1016/j.neuron.2015.12.023 26844829 PMC4742785

[B20] Gianfranceschi L, Siciliano R, Walls J, Morales B, Kirkwood A, Huang ZJ, Tonegawa S, Maffei L (2003) Visual cortex is rescued from the effects of dark rearing by overexpression of BDNF. Proc Natl Acad Sci USA 100:12486–12491. 10.1073/pnas.1934836100 14514885 PMC218784

[B21] Gilley J, Coleman MP (2010) Endogenous Nmnat2 is an essential survival factor for maintenance of healthy axons. PLoS Biol 8. 10.1371/journal.pbio.1000300 20126265 PMC2811159

[B22] Gordon A, Stryker P (1996) Experience-dependent plasticity of binocular responses in the primary visual cortex of the mouse. J Neurosci 76:3274–3286. 10.1523/JNEUROSCI.16-10-03274.1996 8627365 PMC6579137

[B23] Hanover JL, Huang ZJ, Tonegawa S, Stryker MP (1999) Brain-derived neurotrophic factor overexpression induces precocious critical period in mouse visual cortex. J Neurosci 19:RC40. 10.1523/JNEUROSCI.19-22-j0003.1999 10559430 PMC2424259

[B24] Haruta M, Hata Y (2007) Experience-driven axon retraction without binocular imbalance in developing visual cortex. Curr Biol 17:37–42. 10.1016/j.cub.2006.10.064 17208184

[B25] Heimel JA, Hartman RJ, Hermans JM, Levelt CN (2007) Screening mouse vision with intrinsic signal optical imaging. Eur J Neurosci 25:795–804. 10.1111/j.1460-9568.2007.05333.x 17328775

[B26] Hensch TK (2005) Critical period plasticity in local cortical circuits. Nat Rev Neurosci 6:877–888. 10.1038/nrn1787 16261181

[B27] Hensch TK, Fagiolini M, Mataga N, Stryker MP, Baekkeskov S, Kash SF (1998) Local GABA circuit control of experience-dependent plasticity in developing visual cortex. Science 282:1504–1508. 9822384 10.1126/science.282.5393.1504 PMC2851625

[B28] Hofer SB, Mrsic-Flogel TD, Bonhoeffer T, Hübener M (2006) Lifelong learning: ocular dominance plasticity in mouse visual cortex. Curr Opin Neurobiol 16:451–459. 10.1016/j.conb.2006.06.007 16837188

[B29] Hoopfer ED, McLaughlin T, Watts RJ, Schuldiner O, O’Leary DDM, Luo L (2006) Wlds protection distinguishes axon degeneration following injury from naturally occurring developmental pruning. Neuron 50:883–895. 10.1016/j.neuron.2006.05.013 16772170

[B30] Huang X, Stodieck SK, Goetze B, Cui L, Wong MH, Wenzel C, Hosang L, Dong Y, Löwel S, Schlüter OM (2015) Progressive maturation of silent synapses governs the duration of a critical period. Proc Natl Acad Sci USA 112:E3131–E3140. 10.1073/pnas.1506488112 26015564 PMC4475980

[B31] Huang ZJ, Kirkwood A, Pizzorusso T, Porciatti V, Morales B, Bear MF, Maffei L, Tonegawa S (1999) BDNF regulates the maturation of inhibition and the critical period of plasticity in mouse visual cortex. Cell 98:739–755. 10.1016/S0092-8674(00)81509-3 10499792

[B32] Jenks KR, Kim T, Pastuzyn ED, Okuno H, Taibi AV, Bito H, Bear MF, Shepherd JD (2017) Arc restores juvenile plasticity in adult mouse visual cortex. Proc Natl Acad Sci USA 114:201700866. 10.1073/pnas.1700866114 28790183 PMC5576785

[B33] Kerschensteiner M, Schwab ME, Lichtman JW, Misgeld T (2005) In vivo imaging of axonal degeneration and regeneration in the injured spinal cord. Nat Med 11:572–577. 10.1038/nm1229 15821747

[B34] Kuhlman SJ, Olivas ND, Tring E, Ikrar T, Xu X, Trachtenberg JT (2013) A disinhibitory microcircuit initiates critical-period plasticity in the visual cortex. Nature 501:543–546. 10.1038/nature12485 23975100 PMC3962838

[B35] Lehmann K, Löwel S (2008) Age-dependent ocular dominance plasticity in adult mice. PLoS One 3:e3120. 10.1371/journal.pone.0003120 18769674 PMC2518841

[B36] Levelt CN, Hübener M (2012) Critical-period plasticity in the visual cortex. Annu Rev Neurosci 35:309–330. 10.1146/annurev-neuro-061010-113813 22462544

[B37] Lohmann C, Kessels HW (2014) The developmental stages of synaptic plasticity. J Physiol 592:13–31. 10.1113/jphysiol.2012.235119 24144877 PMC3903349

[B38] Lunn ER, Perry VH, Brown MC, Rosen H, Gordon S (1989) Absence of Wallerian degeneration does not hinder regeneration in peripheral nerve. Eur J Neurosci 1:27–33. 12106171 10.1111/j.1460-9568.1989.tb00771.x

[B39] Lyon MF, Ogunkolade BW, Brown MC, Atherton DJ, Perry VH (1993) A gene affecting Wallerian nerve degeneration maps distally on mouse chromosome 4. Proc Natl Acad Sci USA 90:9717–9720. 8415768 10.1073/pnas.90.20.9717 PMC47641

[B40] McGee AW, Yang Y, Fischer QS, Daw NW, Strittmatter SH (2005) Experience-driven plasticity of visual cortex limited by myelin and nogo receptor. Science 309:2222–2226. 10.1126/science.1114362 16195464 PMC2856689

[B41] Morishita H, Hensch TK (2008) Critical period revisited: impact on vision. Curr Opin Neurobiol 18:101–107. 10.1016/j.conb.2008.05.009 18534841

[B42] Pease SE, Segal RA (2014) Preserve and protect: maintaining axons within functional circuits. Trends Neurosci 37:572–582. 10.1016/j.tins.2014.07.007 25167775 PMC4245037

[B43] Pham TA, Graham SJ, Suzuki S, Barco A, Kandel ER, Gordon B, Lickey ME (2004) A semi-persistent adult ocular dominance plasticity in visual cortex is stabilized by activated CREB. Learn Mem 11:738–747. 10.1101/lm.75304 15537732 PMC534702

[B44] Pizzorusso T, Medini P, Berardi N, Chierzi S, Fawcett JW, Maffei L (2002) Reactivation of ocular dominance plasticity in the adult visual cortex. Science 298:1248–1251. 10.1126/science.1072699 12424383

[B45] Pollak N, Dölle C, Ziegler M (2007) The power to reduce: pyridine nucleotides – small molecules with a multitude of functions. Biochem J 402:205–218. 10.1042/BJ20061638 17295611 PMC1798440

[B46] Saiepour MH, Min R, Kamphuis W, Heimel JA, Levelt CN (2017) β-Catenin in the adult visual cortex regulates NMDA-receptor function and visual responses. Cereb Cortex 28:1183–1194. 10.1093/cercor/bhx029 28184425

[B47] Sasaki Y, Araki T, Milbrandt J (2006) Stimulation of nicotinamide adenine dinucleotide biosynthetic pathways delays axonal degeneration after axotomy. J Neurosci 26:8484–8491. 10.1523/JNEUROSCI.2320-06.2006 16914673 PMC6674352

[B48] Sasaki Y, Nakagawa T, Mao X, DiAntonio A, Milbrandt J (2016) NMNAT1 inhibits axon degeneration via blockade of SARM1-mediated NAD+ depletion. Elife 5. 10.7554/eLife.19749 27735788 PMC5063586

[B49] Sato M, Stryker MP (2008) Distinctive features of adult ocular dominance plasticity. J Neurosci 28:10278–10286. 10.1523/JNEUROSCI.2451-08.2008 18842887 PMC2851628

[B50] Sawtell NB, Frenkel MY, Philpot BD, Nakazawa K, Tonegawa S, Bear MF (2003) NMDA receptor-dependent ocular dominance plasticity in adult visual cortex. Neuron 38:977–985. 12818182 10.1016/s0896-6273(03)00323-4

[B51] Siegel F, Heimel JA, Peters J, Lohmann C (2012) Peripheral and central inputs shape network dynamics in the developing visual cortex in vivo. Curr Biol 22:253–258. 10.1016/j.cub.2011.12.026 22264606

[B52] Sommeijer JP, Levelt CN (2012) Synaptotagmin-2 is a reliable marker for parvalbumin positive inhibitory boutons in the mouse visual cortex. PLoS One 7:e35323. 10.1371/journal.pone.0035323 22539967 PMC3335159

[B53] Stephany CE, Chan LLH, Parivash SN, Dorton HM, Piechowicz M, Qiu S, McGee AW (2014) Plasticity of binocularity and visual acuity are differentially limited by Nogo receptor. J Neurosci 34:11631–11640. 10.1523/JNEUROSCI.0545-14.2014 25164659 PMC4145169

[B54] Stephany CÉ, Ma X, Dorton HM, Wu J, Solomon AM, Frantz MG, Qiu S, McGee AW (2018) Distinct circuits for recovery of eye dominance and acuity in murine amblyopia. Curr Biol 28:1914–1923.e5. 10.1016/j.cub.2018.04.055 29887305 PMC6008222

[B55] Syken J, GrandPre T, Kanold PO, Shatz CJ (2006) PirB restricts ocular-dominance plasticity in visual cortex. Science 313:1795–1800. 10.1126/science.1128232 16917027

[B56] Tognini P, Putignano E, Coatti A, Pizzorusso T (2011) Experience-dependent expression of miR-132 regulates ocular dominance plasticity. Nat Neurosci 14:1237–1239. 10.1038/nn.2920 21892154 PMC3183093

[B57] Tremblay MÈ, Lowery RL, Majewska AK (2010) Microglial interactions with synapses are modulated by visual experience. PLoS Biol 8. 10.1371/journal.pbio.1000527 21072242 PMC2970556

[B58] Tropea D, Majewska AK, Garcia R, Sur M (2010) Structural dynamics of synapses in vivo correlate with functional changes during experience-dependent plasticity in visual cortex. J Neurosci 30:11086–11095. 10.1523/JNEUROSCI.1661-10.2010 20720116 PMC2932955

[B59] Vargas ME, Barres BA (2007) Why is Wallerian degeneration in the CNS so slow? Annu Rev Neurosci 30:153–179. 10.1146/annurev.neuro.30.051606.094354 17506644

[B60] Vetencourt JFM, Sale A, Viegi A, Baroncelli L, De Pasquale R, O’Leary OF, Castren E, Maffei L (2008) The antidepressant fluoxetine restores plasticity in the adult visual cortex. Science 320:385–388. 10.1126/science.1150516 18420937

[B61] Wang BS, Sarnaik R, Cang J (2010) Critical period plasticity matches binocular orientation preference in the visual cortex. Neuron 65:246–256. 10.1016/j.neuron.2010.01.002 20152130 PMC2822731

[B62] Yahata N, Yuasa S, Araki T (2009) Nicotinamide mononucleotide adenylyltransferase expression in mitochondrial matrix delays Wallerian degeneration. J Neurosci 29:6276–6284. 10.1523/JNEUROSCI.4304-08.2009 19439605 PMC6665489

